# Geriatric palliative care: a view of its concept, challenges and strategies

**DOI:** 10.1186/s12877-018-0914-0

**Published:** 2018-09-20

**Authors:** R. Voumard, E. Rubli Truchard, L. Benaroyo, G. D. Borasio, C. Büla, R. J. Jox

**Affiliations:** 1 Service of Palliative and Supportive Care, Department of Medicine, Lausanne University Hospital, Avenue Pierre-Decker 5, CH-1011 Lausanne, Switzerland; 2 Geriatric Palliative Care, Department of Medicine, Lausanne University Hospital, Avenue Pierre-Decker 5, CH-1011 Lausanne, Switzerland; 3 Service of Geriatric Medicine and Geriatric Rehabilitation, Department of Medicine, Lausanne University Hospital, Chemin de Mont-Paisible 16, CH-1011 Lausanne, Switzerland; 4 Clinical Ethics Unit and Institute of Humanities in Medicine, Faculty of Biology and Medicine, University of Lausanne, Avenue de Provence 82, CH-1011 Lausanne, Switzerland

**Keywords:** Geriatrics, Palliative care, Interdisciplinary, Health policy, Ethics of care

## Abstract

In aging societies, the last phase of people’s lives changes profoundly, challenging traditional care provision in geriatric medicine and palliative care. Both specialties have to collaborate closely and geriatric palliative care (GPC) should be conceptualized as an interdisciplinary field of care and research based on the synergies of the two and an ethics of care.

Major challenges characterizing the emerging field of GPC concern (1) the development of methodologically creative and ethically sound research to promote evidence-based care and teaching; (2) the promotion of responsible care and treatment decision making in the face of multiple complicating factors related to decisional capacity, communication and behavioural problems, extended disease trajectories and complex social contexts; (3) the implementation of coordinated, continuous care despite the increasing fragmentation, sectorization and specialization in health care.

Exemplary strategies to address these challenges are presented: (1) GPC research could be enhanced by specific funding programs, specific patient registries and anticipatory consent procedures; (2) treatment decision making can be significantly improved using advance care planning programs that include adequate decision aids, including those that address proxies of patient who have lost decisional capacity; (3) care coordination and continuity require multiple approaches, such as care transition programs, electronic solutions, and professionals who act as key integrators.

## Background

The increasing life expectancy and the associated changes in end-of-life morbidity forecast major challenges for health care [1]. Today in Europe, 50-year-old women and men can expect to live 34 and 29 more years, respectively. Yet, the expected time free of morbidity is only 10 and 9 years, respectively [2]. This means that the last two decades of most people’s lives are characterized by an increasing burden of chronic multimorbidity, functional dependency, frailty and often cognitive decline, necessitating a geriatric approach to care [3].

At the same time, the causes of death shift, the dying phase changes, and the last period of life extends to a long phase characterized by complicated treatment decisions, difficult symptom management, manifold psychosocial problems and easily overlooked spiritual distress. Thus, the necessity of hospice and palliative care, tailored to the needs and situations of the elderly and very elderly, is evident [4], especially with regard to the growing number of people who live in residential care homes or assisted living facilities [5, 6]. The emerging field of *geriatric palliative care (GPC)*, while having been pioneered during recent years [7], still lacks a sufficient evidence base. It also needs a broadly accepted definition and a sound conceptual foundation.

With this article, we intend to stimulate the reflection and debate internationally about the evolution of GPC in the years to come. Based on a local effort to bring together expertise in GPC, this paper contributes to the debate by defining the theoretical core concept of GPC with its ethical underpinnings, delineating its major challenges, and sketching some exemplary strategies to address them. Clarifying these questions is important for health care providers and policymakers to steer the development of GPC in the right direction.

## Theoretical foundations and concept of GPC

To elucidate the concept of GPC, we first characterize its three main elements before combining them to offer a working definition of GPC.

### Geriatric medicine

Geriatric medicine is the medical specialty focusing on the health care of elderly persons. It was developed as a response to the multimorbidity of the growing elderly patient population. It focuses on the prevention, assessment and management of their specific health problems across the disease trajectories and includes the physical, mental, social and spiritual dimensions (Table 1). Health complexity is one of the hallmarks of geriatric medicine [8]. The main goals are the maintenance and restoration of functional capabilities, thus improving quality of life and social participation.

**Table 1:** Definition of geriatric palliative care and its relevant elements

Geriatric Medicine	Geriatric Medicine is a specialty of medicine concerned with physical, mental, functional and social conditions in acute, chronic, rehabilitative, preventive, and end-of-life care in older patients. (European Union of Medical Specialists 2008 [43])
Palliative Care	Palliative care is an approach that improves the quality of life of patients and their families facing the problem associated with life-threatening illness, through the prevention and relief of suffering by means of early identification and impeccable assessment and treatment of pain and other problems, physical, psychosocial and spiritual. (World Health Organization 2004 [1])
Ethics of Care	The ethics of care builds relations of care that highlight the deeper reality of human interdependency and the need for caring to surround liberal autonomy. It provides a way of reflection in order to develop morally acceptable human relations and societies. (Adapted from “The Ethics of Care”, Oxford 2006 [44])
Geriatric Palliative Care	Geriatric Palliative Care integrates the complementary specialties of geriatrics and palliative care to provide comprehensive care for older patients entering the later stage of their lives, and their families. (adapted from “Geriatric Palliative Care”, Oxford 2014 [20])

Within the general care for older people, geriatrics offers specialized coordinated care for often very old patients. Most patients will be over 65 years of age, but the problems best dealt with by the specialty become much more common in the 80+ age group. These patients often have a high degree of frailty and multiple active chronic pathologies [9]. Geriatrics makes use of multidimensional and interdisciplinary assessments and can be considered a meta-discipline [10]. It promotes the collaboration across multiple health care settings, ideally constructing the care approach around the patient’s problems and preferences.

### Palliative care

In contrast to geriatrics, palliative care is a specialty that applies to patients of all ages, but with special needs linked to dying in a very broad sense. Modern palliative care, understood in its broadest sense that also includes hospice care, evolved 50 years ago out of three sources: (1) the critical societal climate in the 1960s that challenged the taboo surrounding death and dying [11]; (2) a reform movement within health care that attacked the technological imperative of medicine, which neglected the dying and incurably ill [12]; and (3) a religiously influenced emphasis on professional virtues like caring, compassion, and empathy [11].

Similar to geriatrics, palliative care is grounded in a holistic anthropology, integrating on the same level the physical, psychological, social, and spiritual dimensions of the human being, which is mirrored in a multiprofessional team approach. In caring for patients suffering from severe and life-threatening illness up until death, palliative care aims to improve quality of life and ease suffering by preventing and treating symptoms instead of diseases (Table 1). A further characteristic is the idea of the unit of care, embracing both the patients and their significant others, who are recognized not only as caregivers and substitute decision makers, but also as persons in need of support.

### Ethics of care

For the elderly patient population with palliative care needs, the ethical approach that is particularly apt is the ethics of care (Table 1). It complements the traditional normative ethics, which is primarily based on action principles and individual autonomy. The ethics of care regards the patient’s vulnerability as the source of a context-sensitive, prudential judgment and care. Vulnerability is conceived not just as a lack of autonomy, but also as a call for the health care professional to strengthen the patient’s capabilities. Autonomy itself is understood as relational autonomy constituted and enriched by interpersonal relationships.

When professional caregivers face old and frail people who are severely ill or dying, one of their major tasks is to articulate perspectives and open a space of dialogue, taking into consideration the patients’ and their loved ones’ narratives [13]. This approach can help build a relation of trust that empowers patients to continuously reframe their identity, formulate life plans and set goals of care. This is all the more important in the context of restricted freedom of agency, dependence in activities of daily living, social isolation, cognitive impairment, chronic suffering or imminent death.

### Geriatric palliative care

**Fig. 1:**
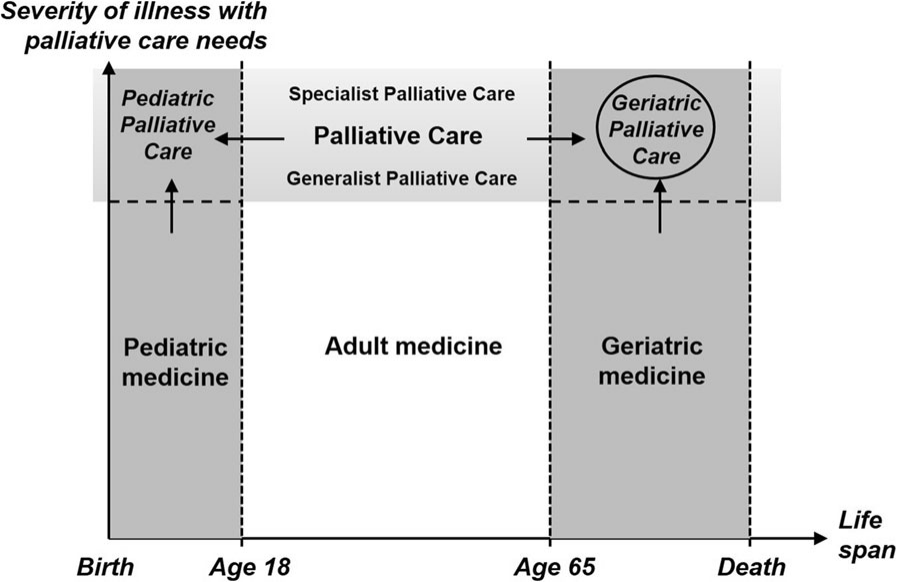
Place of geriatric palliative care in the context of both geriatric medicine and palliative care. The dotted lines symbolize transitions where a clear-cut border cannot be drawn. Palliative care may begin prenatally and includes post mortem family bereavement. Palliative care includes both specialist-level and generalist-level care

Building upon these three elements, GPC can be understood as an approach that aims to improve the quality of life of elderly persons facing severe and life-threatening illness near the end of their lives. While geriatrics is defined by the life period of its patient population and palliative care by its particular goals of care, GPC is not situated at the same level: it is neither a new specialty nor a subspecialty within these two, but rather an inter-specialty collaboration at the intersection of geriatrics and palliative care (Fig. 1).

Geriatrics and palliative care are distinct but overlapping medical specialties [14, 15]. They are both highly multiprofessional and interdisciplinary fields with patient- and family-centred activities aimed at improving quality of life, personal capabilities and social participation [16]. The synergies that result from joining these related specialties may serve as a role model for inter-specialty collaboration in health care. In today’s hyper-specialized and increasingly fragmented medical world, we need an integrative approach that zooms out to the global picture of the patient’s life situation. While integrated care and continuity of care models are important on the level of the health care providers [17, 18], we also need a closer collaboration of the professional specialties geriatrics and palliative care, e.g. by organizing inter-specialty continued education. Thus, the field of GPC may be able to offer a profoundly integrated care that may encompass different goals of care but that also facilitates a sound process of shifting from the goal of functional recovery to purely comfort-oriented goals [19].

### Some major challenges of GPC

An exhaustive review of the current state of GPC is beyond the scope of this article. Among the multiple challenges of GPC we would like to select three pressing ones [20]. The first challenge is to conduct methodologically sound and ethically justified research to offer evidence-based interventions of care and training. Pharmacological trials usually exclude multimorbid geriatric patients, which limits the applicability of their results in this population. Severely ill elderly persons have the same right to be included in research as all other patients, yet studies are methodologically difficult due to many patients’ cognitive problems (complicating informed consent), gatekeeper effects, and high drop-out rates in view of the short life expectancy.

Another major challenge is making health care decisions for the severely ill elderly, both at the end of life and in anticipation thereof. Seventy percent of patients over the age of 60 for whom end-of-life decisions have to be taken do not have full decisional capacity [21]. The decision-making process is complicated by multiple factors: communication barriers, deficits in cognition and recollection necessitating a reconstruction of the patient’s narrative and personal values, the tension between patients’ and their proxies’ interests, and the difficulty to interpret non-verbal behavior of patients who lack decision-making capacity [22]. Caregivers who decide on behalf of the patients must use prudential judgment, avoiding the twin pitfalls of ageist undertreatment and futile overtreatment. The instruments hitherto employed to ensure care consistent with the patient’s preferences are far from ideal: traditional advance directives are not as effective as hoped, and surrogate decision makers often err widely in their substituted judgment [23].

As care trajectories of older patients are usually long and characterized by multiple transitions between health care settings, a third challenge is the coordination of care. The lack of coordination is a major cause of wasted resources, weakening the health system and reducing quality of care [24]. A growing imbalance exists between the multitude of specialists and the lack of care continuity. Exaggerated polypharmacy and conflicting recommendations sometimes put the patient more at risk than the disease itself [25]. The large body of evidence on burdensome interventions, hospitalizations and emergency departments visits in the last months of the lives of the elderly also points to failures in care coordination [26–28]. In many countries, home-based palliative care provision is particularly underdeveloped and contributes to the shift toward the inpatient sector at the end of life [29, 30].

### Exemplary strategies in GPC

These three selected challenges of GPC are best addressed in a joint effort. In order to boost clinical research in this area, public recognition is needed, expressed by specific funding programs, academic endeavours, and public knowledge transfer. Patient registries could be used to study the natural course of the last phase of life in old age and the related needs of patients and their families. Approaching patients when they still possess decisional capacity may allow them to use anticipatory research consent or to instruct relatives so that they will be able to give a well-founded proxy consent later on. Palliative care needs of older patients have been found to be different from those of younger ones [5], so we also need specific interventional studies addressing these needs. Up to now, however, the number of high-quality effectiveness studies in GPC is low [31, 32]. Cluster randomized controlled trials are currently underway [33] and they intend to prove a similar level of effectiveness as already exists for palliative care in general [34]. In parallel, we need to do more research on appropriate quality of care measures for this particular population with its specific needs [29].

The challenges in healthcare decision making could be met by effective decision aids and advance care planning (ACP), a comprehensive communication approach that ensures adequate documentation and implementation of patient preferences [35]. Programs like “Respecting Patient Choices” in Australia have shown their potential to increase patient-centered care, reduce distress in patients and families, and the quality of end-of-life care [36, 37]. In applying ACP to the GPC population, it may be necessary to develop programs tailored to the needs of patients with progressive cognitive impairment. For the multitude of GPC patients who have already lost their decision-making capacity, it is up to the patients’ proxies to engage in an ACP conversation with health care professionals, exclusively oriented towards patients’ preferences (called "ACP by proxy") [38].

Lastly, poor care coordination can and should be tackled at a variety of places. It starts with coordinating inpatient care for seriously ill elderly [39, 40], includes discharge planning, liaison services and care transition programs, and indispensably requires community palliative care coordination for the elderly, both in nursing homes and at home [30, 41]. A major support could be the use of new technologies, such as electronic documentation and telemedicine. This could allow a more effective sharing of information between the different stakeholders. In addition, the coordination between various healthcare professionals will be more successful if we develop, already in the qualification phase, a truly interprofessional culture in healthcare, specifically in GPC. Moreover, so-called key integrators are needed (e.g. general practitioners, nurse practitioners, case managers) who follow the patients over a long period of time and are able to manage and integrate the different aspects of health care. They could also be central chaperons to orchestrate and facilitate the important ACP process mentioned above. Care coordination demonstrably enhances quality of life, limits treatment-related harm and saves unnecessary costs to be reallocated for the real benefit of patients [42].

## Conclusions

The goal of GPC is to develop and offer evidence-based management strategies for the elderly population with severe and life-limiting conditions. Major challenges in this area include establishing high-quality, ethically sound research projects in this vulnerable patient population, facilitating responsible healthcare decision making, and ensuring good coordination of care. We sketched several strategies to address these problems, with a focus on the all-important issue of ACP.

Finally, it is central to promote practical wisdom in professionals to deliver care that responds to the needs and expectations of the patients and their families. As severely ill elderly persons constitute a highly vulnerable group, their wellbeing depends on care that is multidimensional, sustainable and oriented towards relational autonomy. In this phase of life, close relationships of trust become decisive, and this bears on the attitudes and responsibilities expected from professional caregivers.

## Summary

- Geriatric Palliative Care (GPC) is a field of inter-specialty collaboration unifying competences from geriatric medicine and palliative care to respond to the socio-demographic changes and challenges of older adults with severe and life-limiting conditions.
- Main challenges of GPC include clinical research in frail and cognitively impaired patients, healthcare decision making including advance care planning, and coordination of care.
- An approach based on the ethics of care and practical wisdom is required in order to help health care professionals deliver care that responds to the needs of this particularly vulnerable group of patients and their families.

## Abbreviations

ACP: Advance Care Planning
GPC: Geriatric Palliative Care
